# Corvid Re-Caching without ‘Theory of Mind’: A Model

**DOI:** 10.1371/journal.pone.0032904

**Published:** 2012-03-01

**Authors:** Elske van der Vaart, Rineke Verbrugge, Charlotte K. Hemelrijk

**Affiliations:** 1 Institute of Artificial Intelligence, University of Groningen, Groningen, The Netherlands; 2 Behavioural Ecology and Self-Organisation, University of Groningen, Groningen, The Netherlands; Cajal Institute, Consejo Superior de Investigaciones Científicas, Spain

## Abstract

Scrub jays are thought to use many tactics to protect their caches. For instance, they predominantly bury food far away from conspecifics, and if they must cache while being watched, they often re-cache their worms later, once they are in private. Two explanations have been offered for such observations, and they are intensely debated. First, the birds may reason about their competitors' mental states, with a ‘theory of mind’; alternatively, they may apply behavioral rules learned in daily life. Although this second hypothesis is cognitively simpler, it does seem to require a different, ad-hoc behavioral rule for every caching and re-caching pattern exhibited by the birds. Our new theory avoids this drawback by explaining a large variety of patterns as side-effects of stress and the resulting memory errors. Inspired by experimental data, we assume that re-caching is not motivated by a deliberate effort to safeguard specific caches from theft, but by a general desire to *cache more*. This desire is brought on by stress, which is determined by the presence and dominance of onlookers, and by unsuccessful recovery attempts. We study this theory in two experiments similar to those done with real birds with a kind of ‘virtual bird’, whose behavior depends on a set of basic assumptions about corvid cognition, and a well-established model of human memory. Our results show that the ‘virtual bird’ acts as the real birds did; its re-caching reflects whether it has been watched, how dominant its onlooker was, and how close to that onlooker it has cached. This happens even though it cannot attribute mental states, and it has only a single behavioral rule assumed to be previously learned. Thus, our simulations indicate that corvid re-caching can be explained without sophisticated social cognition. Given our specific predictions, our theory can easily be tested empirically.

## Introduction

Over the last decade, the social cognition of corvids – the extended family of crows – has been the subject of much scientific attention. Experiments have shown, for instance, that Clark's nutcrackers can use human cues to find food [1], that pinyon jays can reason about social hierarchies [2], and that rooks can cooperate to obtain rewards [3]. Most impressive are the behaviors that ravens [4], [5], [6], [7] and scrub jays [8], [9], [10], [11], [12], [13] display in the context of caching. Like most corvids, these species hide food under ground, saving it for later. However, items may be stolen by conspecifics that saw the caching occur. This could create an incentive for the birds to be sensitive to the visual perspectives of others [14], and many results appear to confirm that they are. When pilfering, if two ravens are present at a caching event, the more subordinate one pilfers faster if the cache site was within the dominant one's field of vision, and thus likely to be stolen, than if the cache site was not [5]. Similarly, when a raven is shown two cache sites in front of a competitor, it first raids the cache that the competitor also had a line of sight to, and only then the other [7]. When caching, corvids bury most of their items far away from onlookers, and behind barriers [4], [11], [12]. Furthermore, scrub jays often re-cache their worms later, when they are in private, if they were forced to cache in the presence of others [8], [9], [10], [11], [12].

The two explanations that have been offered for these results are the subject of intense debate [14], [15], [16], [17]. First, the birds could be reasoning about the mental states of their competitors [14]. A scrub jay might infer that other birds *intend* to steal its worms, and that if others *see* it caching they will *know* where its worms are. Furthermore, a scrub jay could realize that caching far away from onlookers makes it difficult for them to *see* its caches, and that re-caching when alone will ensure that they no longer *know* the locations of its items. According to this hypothesis, scrub jays thus have some elements of a ‘theory of mind’. Alternatively, the birds could be applying behavioral rules that they have learned previously, from experience in daily life [16]. For instance, through cache interruptions, ravens could learn the rule ‘cache far away from onlookers’ [18]: The nearer conspecifics are, the greater the likelihood that one of them will try to take the food the cacher is trying to bury. In this way, the birds could learn that the proximity of conspecifics implies cache loss, and should therefore be avoided. However, they might also learn rules that are more complex; for example, they might associate ‘a specific competitor's line of sight in the past’ with ‘a general feeling of unease’ regarding a particular cache site.

A reason to favor this ‘prior learning hypothesis’ is that it seems cognitively simpler than ‘theory of mind’; it does not require corvids to be capable of mental state attribution, which is a controversial claim for all species other than humans [14], [15], [16], [17]. However, as Tomasello and Call [19] have argued, a weakness of current accounts that depend on prior learning is that almost every experimental result has to be explained by its own ad-hoc behavioral rule, acquired in some hypothetical past situation; see, for instance, Penn and Povinelli [16]. Furthermore, for some of the more complex re-caching patterns exhibited by scrub jays, it is difficult to imagine prior learning scenarios that are plausible. For instance, in two studies [11], [12], the birds not only preferred to cache far away from conspecifics; hours later, when they were alone, they also re-cached more of what they had cached close to other birds. To learn this, the birds would have to remember the distances between cache sites and onlookers, and also relate these distances to pilfering rates. This seems cognitively complex, especially for scrub jays in small aviaries [20], where the effect of distance is likely to be small, and thus, difficult to detect.

Therefore, our aim is to develop a cognitively simple theory that can explain the re-caching behavior of scrub jays without these drawbacks. We do this by taking our existing computational model of corvid cognition, as we have already shown that it generates caching behavior that resembles that of real corvids [21]. In the present paper, we extend this model with a single behavioral rule assumed to be previously learned – a preference for caching far away from conspecifics – and a new set of assumptions related to *stress*. The model consists of a ‘virtual bird’. Its basic behavior is driven by a *memory system*, based on broadly validated, similar models built for humans [22], [23]; for previous applications to other species, see [24], [25]. It stores one integrated memory of every act of caching and recovery [26], and the more often, and the more recently, it has cached or recovered at a particular site, the stronger that memory will be [22]. The stronger its memory of having cached somewhere, the lower its tendency to cache there again, and the higher its tendency to recover there later. Similarly, the stronger its memory of having recovered somewhere, the lower its tendency to recover there again.

With regards to our model extensions related to *stress*, we assume that stress causes *increased caching* and, as a special case, *increased re-caching*. This is inspired by work explicitly linking stress to caching [27], based on the observation that birds cache more when faced with poor habitat quality [28], [29], [30], [31] and light body weight [32], [33], [34]. Along the same lines, we interpret it as a sign of stress that scrub jays often cache [8], [10] and re-cache [11] more when watched. Such enhanced caching in the presence of conspecifics has also been reported for Eurasian jays [35], although the opposite pattern has also been found, for both Eurasian jays [36] and other species of corvid [37], [38]. Here, we focus on scrub jays, and thus, to mimic their behavior, we make our ‘virtual bird’ re-cache more when it is watched. We also make it re-cache more if the spectator is dominant than if it is subordinate, as we assume that the former evokes more stress. Furthermore, we posit that, in recovery sessions, *finding caches missing* is a source of stress, and this, accordingly, makes our ‘virtual bird’ re-cache more.

We investigate the consequences of these assumptions by exposing our ‘virtual bird’ to simulated versions of two experiments. The first compares caching in front of a conspecific to caching alone, as done in an experiment by Emery and Clayton [8]; the second concerns the effects of distance and the dominance of onlookers, as tested by Dally, Emery and Clayton [12]. We show that in both cases, our ‘virtual bird’ acts as the real birds did.

## Results

The experiments that we simulated consisted of one *caching session* and one *recovery session*, in which the scrub jays were tested individually, in their home cages [8], [12]. In a caching session, the scrub jays were given a bowl of worms, and ice cube trays to cache in. After caching, both were removed. The birds were left without any food until the recovery session, several hours later. At that point, they were mildly hungry, and their trays were returned to them. For each experiment, we ran one hundred simulations at the original sample size, and we created as many ‘virtual birds’ as there were real birds. We averaged the results for each run, and calculated the discrepancy with the empirical data. Further analysis was done on the run closest to the average discrepancy. The model has three adjustable parameters (Table S1): *d*, governing how quickly memories decay, *n*, specifying the noise in their likelihood of recall, and *st*, determining the stress threshold at which recovering ‘virtual birds’ re-cache. They were kept constant across experiments, and set so that the performance of the ‘virtual birds’ matched that of the real birds. Our results were robust, and depended only mildly on the model's exact parameter values; see Figure S1, Table S2, and Text S1.

### Experiment 1: Watched by a Conspecific

**Figure 1:**
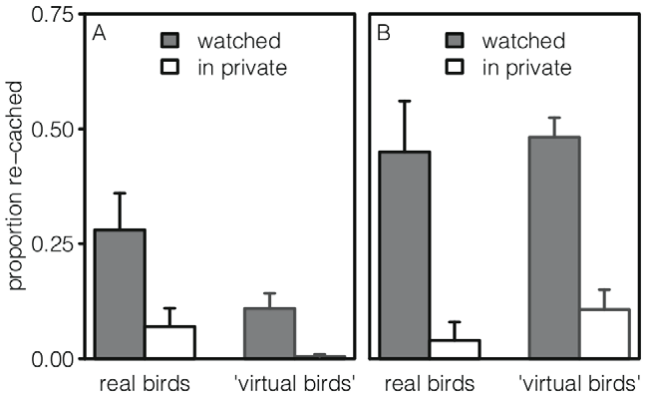
Re-Caching in Experiment 1. Average proportion of caches re-cached, real birds [8] and ‘virtual birds’, with standard errors. Panel A: Alternating ‘watched’ and ‘in private’ trials. Panel B: At recovery, ‘watched’ and ‘in private’ trays presented together.

Emery and Clayton [8] investigated whether scrub jays change their re-caching behavior in response to being watched at caching. This was tested in two different ways. First, the birds were given three trials ‘with onlooker’ and three trials ‘in private’. On ‘with onlooker’ trials, the cachers could see a bird in an adjacent cage; on ‘in private’ trials, the cachers could not. At recovery, they were always in private. The result was that the scrub jays re-cached a significantly larger proportion of their worms on the ‘with onlooker’ trials than the ‘in private’ ones (Figure 1A). In a second setup, the same birds received two trials where they could cache in two trays, one after the other. While caching in one of the trays, there was a conspecific present; while caching in the other, there was not. Several hours later, both trays were returned simultaneously, and the birds could recover in private. It was found that they re-cached a significantly larger proportion of their worms from the ‘with onlooker’ tray than the ‘in private’ tray (Figure 1B).

These results seem to suggest that the scrub jays remembered whether they had been watched or not, and then re-cached in order to protect themselves from future theft [8]. However, our ‘virtual birds’ behaved similarly, without recalling the social context (Figure 1). The ‘virtual birds’ re-cached more on the trials where they had been watched during caching than on the trials where they had not been (Wilcoxon matched-pairs test, *n* = 7, *V* = 0, *p* = 0.03), and they re-cached more from the tray in which they had cached with an onlooker, than from the tray in which they had cached alone (Wilcoxon matched-pairs test, *n* = 7, *V* = 0, *p* = 0.02). In the model, this is due to memory errors. In the caching session, watched ‘virtual birds’ already re-cached, because they were stressed by the presence of the spectator. Consequently, in the recovery session, they remembered caching in many more sites than actually contained worms. This confusion of their memory caused them to experience more failed recovery attempts, which in turn caused them to be more stressed – and thus, to re-cache more.

### Experiment 2: Onlooker Distance and Social Status

**Figure 2:**
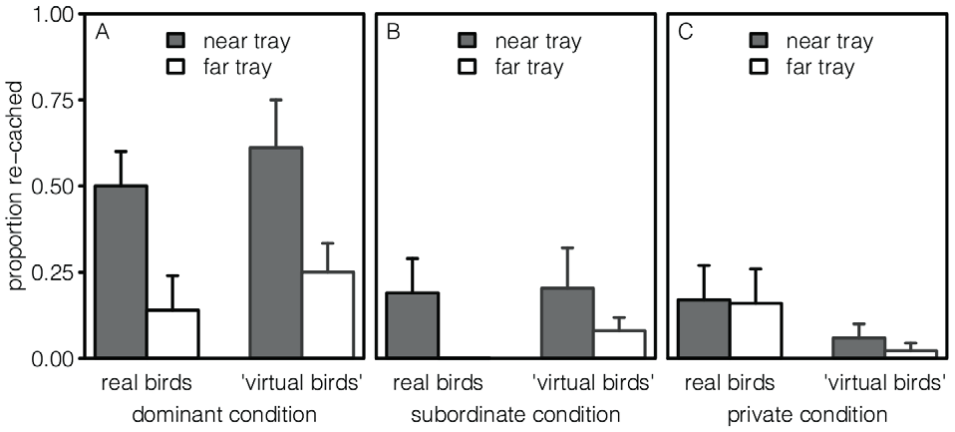
Re-Caching in Experiment 2. Average proportion of caches re-cached per tray, with standard errors, real birds [12], [52] and ‘virtual birds’, with standard errors, after caching in front of a dominant onlooker (Panel A), a subordinate onlooker (Panel B), or in private (Panel C).

Dally, Emery and Clayton [12] investigated whether scrub jays could take into account the proximity of another bird, as well as its social status. To test this, the birds were allowed to cache in three different conditions: Either in front of a dominant onlooker, in front of a subordinate onlooker, or in private, with an opaque partition separating them from a neighbor. In each case, they were offered two ice cube trays to cache in, one near the adjacent bird, and one farther away. It was found that the cachers preferred to cache in the far tray when watched - either by a dominant or by a subordinate - but that they showed no preference when alone. Furthermore, during the recovery session, they re-cached a greater proportion of their worms in the ‘dominant onlooker’ condition than in either of the other two. When they had been watched, the birds also seemed to re-cache specifically from the ‘near’ tray (Figure 2A, 2B), although the sample size was too small for statistical analysis.

These results seem to suggest that the re-caching behavior of the birds depended on their memory of the social status of the onlooker, but our ‘virtual birds’ remembered only the locations of cache sites. Nevertheless, like in the empirical data, we found a difference in re-caching rates between conditions (Friedman's analysis of variance, n = 9, χ_2_^2^ = 11.67, p<0.01). This was because the ‘virtual birds’ re-cached more when they had been watched by a dominant than by a subordinate (Wilcoxon matched-pairs test, *n* = 9, *V* = 28, *p* = 0.02), or than when they had been alone (Wilcoxon matched-pairs test, *n* = 9, *V* = 0, *p*<0.01).

This occurred because we made our ‘virtual bird’ more stressed by dominant onlookers than by subordinate ones, and this caused it to re-cache more during the caching session with the dominant onlooker. This increased re-caching during the *caching session* caused its memory to be more confused during the *recovery session*, which in turn caused it to experience more recovery failures. In our model, such recovery failures cause stress, and stress causes re-caching, so this caused the ‘virtual bird’ to re-cache more during the recovery session as well. Furthermore, like the real birds, the ‘virtual bird’ re-cached proportionally more from the ‘near’ tray when it had been watched (Figure 2A, 2B), but not when it had been in private (Figure 2C). This was due to the fact that it avoided the proximity of others at *caching*, which we assume to have been learned from daily life. As a consequence, the likelihood of successfully recovering from the ‘near’ tray was statistically smaller, because there were fewer worms in it. Therefore, the ‘virtual bird’ experienced more recovery failures in the ‘near’ tray, which caused a higher level of stress to be associated with that tray, and thus more re-caching.

## Discussion

The re-caching behavior of our ‘virtual birds’ was similar to that of the scrub jays. However, the ‘virtual birds’ lacked ‘theory of mind’, and had only a single behavioral rule that was assumed to be due to prior learning: A preference for caching far away from conspecifics. In the recovery session, the ‘virtual birds’ did not remember who had watched them, nor how close cache sites had been to an onlooker, nor whether they had been watched at all. Nevertheless, they displayed various behaviors typically interpreted as indicators of ‘cache protection’: They re-cached more after being watched than after being alone, they re-cached more after caching with a dominant conspecific than after caching with a subordinate one, and they re-cached a larger proportion of the worms cached closer to an onlooker than of the worms cached farther away. To summarize, these results can be explained as follows: The more the ‘virtual bird’ was stressed at caching, the more it re-cached during the caching session, and the more its memory was confused later, during the recovery session. The more its memory was confused at recovery, the more often it expected to find worms in sites that were empty; the more it experienced such recovery failures, the more stressed it was, and the more it re-cached. Similarly, the less it had cached in a particular tray, the lower its likelihood of successfully recovering there; the more it failed, the more stressed it was, and the more it re-cached.

### Empirical Predictions

One beneficial aspect of simulation models is that they can generate empirical predictions that can be tested easily [39], [40]. We list four. First, we predict that birds that re-cache more during the recovery session must have also re-cached more during the caching session. After all, it is this re-caching during the caching session that causes the ‘virtual birds’ to experience memory confusion at recovery, and it is the memory confusion that, through stress, causes the re-caching; thus, without increased re-caching during the caching session, our whole explanation breaks down. For the vast majority of experiments, its presence or absence is not reported [8], [9], [10], [12]. Second, we predict that in a recovery session, the birds start re-caching only after a number of recovery failures. If that is not the case, then it cannot be stress from recovery failures that causes re-caching. Third, we predict that scrub jays must always re-cache proportionally more from emptier trays. So, if scrub jays were forced to cache more worms in one tray than in another, they should also re-cache more from the emptier tray. Finally, we predict that *any* cause of stress should produce enhanced re-caching, irrespective of the social context. For instance, if some of a bird's caches were removed by an experimenter before its tray was returned, then we predict that it should re-cache more. Although experiments that include cache removal have frequently been done [26], [41], [42], [43], whether this causes the scrub jays to re-cache at recovery is not reported.

### Other Experiments on the Social Cognition of Western Scrub Jays

Other experiments on the social cognition of scrub jays can also be interpreted within our framework. For instance, the fact that scrub jays use shadows [9] and barriers [11] to protect their caches can be captured by a slight rephrasing of the rule ‘prefer to cache far away from onlookers’ to the more general version ‘prefer to cache where onlookers are difficult to see’, and this can also be assumed to have been previously learned. Then, like in our current simulations, selective re-caching from ‘riskier’ trays would be due to the stress caused by those trays containing fewer worms. Other results require additional rules to be added to our model. For instance, scrub jays seem to take into account which caches have been seen by which onlookers [12]. Thus, it seems that they *do* remember who was present during caching, unlike our ‘virtual birds’. However, this does not imply that they have ‘theory of mind’. Instead, it could be that the onlooker's presence triggers the subject's memory of the stress it felt during caching, and that this causes it to re-cache the associated caches, without any specific intent to prevent *those* worms from being stolen by *that* onlooker. With regards to scrub jays, one final result to consider is the fact that only experienced pilferers appear to re-cache. This has been described as a case of experience projection, ‘it takes a thief to know a thief’ [8]. An alternative explanation is that scrub jays usually do not feel threatened by onlookers in neighboring cages, because such onlookers cannot actually reach their worms. In that case, maybe only birds that have pilfered find it stressful to be watched by an adjacent bird, because only they have experienced that trays can be moved between cages. Thus, our hypothesis that stress drives re-caching can also be extended to account for this result.

### Why Cache More While Being Watched?

Although our model is built upon the observation that Western scrub jays often cache more when conspecifics are present [8], [10], [11], it is still an open question why they do so, especially given that other species of corvid usually show the opposite pattern [36], [37], [44], [45]. Clark's nutcrackers, for instance, have been shown to cache less when being watched than when not [44]. Clary and Kelly [44] speculate that this species difference might be due to the fact that scrub jays are more social than Clark's nutcrackers, and thus might consider the task to be cooperative. However, it seems unlikely that this is the case; if scrub jays consider the task to be cooperative, it is difficult to explain why they prefer to cache far away from conspecifics [11]. Alternatively, one could argue that for social birds it is not always feasible to inhibit caching until alone; they might have to settle for ‘compensating’ future cache theft, rather than avoiding it. Whether birds employ one strategy or the other – ‘cache more while watched’ or ‘cache less while watched’ – might also depend on individual experience, and the specific situation. This would explain why both enhanced [35] and reduced [36] caching have been found for Eurasian jays, and corresponds well with the observation that ravens gradually acquire some of their ‘cache protection techniques’ during development [18].

### The Larger Debate

In terms of the larger debate on the social cognition of corvids, our results offer a way to explain the re-caching behavior of scrub jays without ‘theory of mind’, and without ‘prior learning’ of many different behavioral rules. Importantly, our account captures three different re-caching patterns – based on onlooker presence, dominance and distance – within a single explanatory framework. Thus, our account of corvid re-caching avoids Tomasello and Call's [19] objection to ‘less cognitive’ theories of chimpanzee social behavior – that there seem to be so many of them, with a different ad-hoc rule explaining every single result. Of course, our simulations do not imply that scrub jays are *not* reasoning about the mental states of others; only that such reasoning is not necessary to produce the results of these specific experiments. That leaves many other aspects of corvid cognition to be explored by our model, even if we confine ourselves to studies of caching. Within the social realm, we could focus on the flexible pilfering strategies of ravens [5], [6], [7], or the suppressed caching of Clark's nutcrackers in the presence of conspecifics [44]. Beyond that, there are intriguing results on ‘mental time travel’ in Western scrub jays [46], [47], [48], magpies [49] and Eurasian jays [50]; these three species appear able to recall the ‘what-where-when’ of specific past events [48], [49], and to plan for specific future desires [46], [47], [50]. These are experiments we hope to address in future work.

## Methods

**Figure 3:**
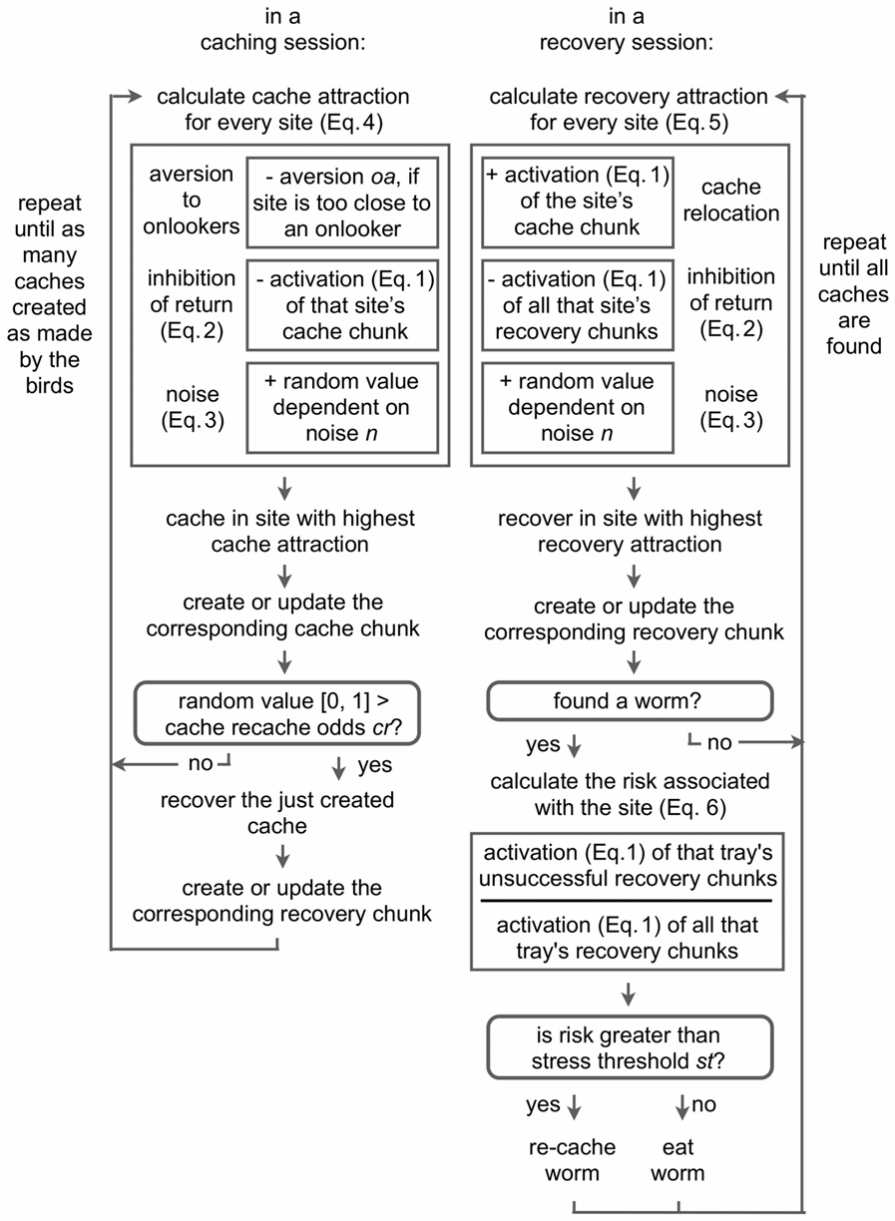
Model flowchart.

To compare our simulations to the behavior of real birds, we use the same statistical tests as in the empirical works [6], [8]. Alpha is set at 0.05, and all tests are two-tailed. Our simulations are implemented in a Java program, *CogCor*, which is included in the Supporting Information, Model Code S1. Conceptually, the program consists of a *setup model*, a *simulator model*, and a *cognitive model*. The setup model keeps track of the state of the ‘physical world’ in the original experiments: What ice cube trays are available, how many worms are cached where, and so on. The simulator model runs the experiments: It ensures that the cognitive model and the setup model are initialized, that the right number of caching and recovery sessions are conducted, and that data is collected for further analysis. The cognitive model is the ‘virtual bird’; it consists of *behavioral rules* (Figure 3), which determine how decisions are made, and *memory chunks*, on which decisions are based.

### Memory Chunks

All caching and recovery events are explicitly encoded in memory, in *chunks*. A chunk's *type* refers to whether it was a caching or recovery event, and its *location* refers to the associated cache site. In the case of a recovery event, a chunk also records *success*, which refers to whether or not a cache was actually found. Thus, a chunk is a memory of a particular kind of *experience* – caching in a site, successfully recovering there, or unsuccessfully recovering there. Every time a ‘virtual bird’ experiences one of these events, it creates the appropriate chunk, and encodes it in memory. If the appropriate chunk already exists, it receives an *update* instead. These updates help determine its *activation*, or ‘memory strength’. A chunk *h*'s activation *A_h_* depends on its *recency* and *frequency* of use [22], as specified by Equation 1, where *t_j_* represents the elapsed time *t* since use *j* of chunk *h*, and *d* is a decay parameter (Table 1). This equation is adapted from ACT-R, a computational model designed to study human cognition [23], [51].
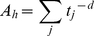
(1)For the purpose of computing the activations of chunks, time is measured in steps. Every cache or recovery event counts as one step, and time *outside* of the experimental sessions is not considered. Although real animals definitely experience memory loss over time, our approach still seems reasonable: The recovery accuracy of Western scrub jays appears to decrease only after retention intervals of several days [42], not the several hours used in these experiments.

**Table 1:** Model variables.

Fixed Settings					Parameters		
*oa_d_*	*oa_s_*	*cr_w_*	*cr_d_*	*cr_s_*	*d*	*n*	*st*
−1.25	−0.5	0.6	0.8	0.4	0.2	0.3	0.6

### Behavioral Rules

The ‘virtual birds’ have two sets of behavioral rules: One for *caching sessions*, one for *recovery sessions*. However, in each case, they must repeatedly decide *where* to cache or recover, and this process is always the same. First, a ‘virtual bird’ evaluates all its possible options – all the discrete ice cube tray sections that are on offer. For each of these options, it estimates the ‘attractiveness’ of caching or recovering there. In caching sessions, it estimates ‘cache attractiveness’, in recovery sessions, it estimates ‘recovery attractiveness’; these will be explained further in coming sections. However, both types of attractiveness always contain at least two components, namely, *inhibition of return*, which helps the ‘virtual bird’ avoid revisiting the same sites with the same purpose, and *noise*.

#### Inhibition of Return

To calculate the inhibition of return *I_k_* associated with a site *k*, a ‘virtual bird’ checks whether any chunks *l* exist of the current session's type, that refer to the same site. If any such chunks *l* exist, the effect of inhibition of return, *I_k_*, associated with site *k* is equal to Equation 2, where ∑*A_l_* is the sum of the activations of all such chunks *l*. Thus, the stronger a ‘virtual bird's’ memories of having cached in a particular location, the lower its tendency to cache there again; similarly, the stronger its memories of having recovered in a particular location, the lower its tendency to recover there again.
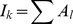
(2)


#### Noise

Every site's attractiveness always has a *noise* component, representing sources of transient error. The *noise* term is computed according to Equation 3, taken from the cognitive architecture ACT-R [23], [51], where *n* is a parameter that we tune (Table 1), and *r* is a random value between 0 and 1.
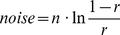
(3)


#### Caching Sessions

In a caching session, a ‘virtual bird’ makes as many caches as the real birds did in the corresponding experiment. Every time it must decide where to cache, it chooses the site with the highest ‘cache attractiveness’ *C_k_*, as determined by Equation 4. Here, *O_k_* stands for the influence of *onlooker aversion*, while *I_k_* refers back to the inhibition of return of Equation 2, and *noise* to the transient error of Equation 3.
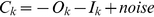
(4)The onlooker aversion component *O_k_* causes the ‘virtual birds’ to avoid caching in the ‘near’ tray in Experiment 2, where they are watched by dominant and subordinate conspecifics [12]. As we assume that increased distance is a preference that the scrub jays have learned before the experiment, we incorporate it directly into the model. Therefore, *O_k_* is equal to *oa_d_* for sites in the ‘near’ tray in the ‘dominant onlooker’ condition, to *oa_s_* for the same sites in the subordinate onlooker condition, and to zero otherwise (Table 1). The settings *oa_d_* and *oa_s_* are tuned so that approximately 25% of the caches end up in the ‘near’ tray in each condition, as found in the empirical data [12].

To capture our assumption that scrub jays are stressed by having to cache in front of a conspecific, and that stress causes them to re-cache [11], watched ‘virtual birds’ can immediately recover a cache they have just created, with odds *cr_w_*. Thus, every time a ‘virtual bird’ caches in front of an onlooker, it has a small chance of immediately recovering its worm. We set *cr_w_* to 0.6, which is tuned to our first experiment. There, it causes the ‘virtual birds’ to re-cache worms an average of 1.29 times when they are watched; this is very close to the empirical average of 1.2 [11]. Furthermore, for Experiment 2, we assume that dominant onlookers evoke more stress than subordinate onlookers, and that this translates into more stress; therefore we set the respective re-caching odds *cr_d_* and *cr_s_* to 0.8 and 0.4 (Table 1).

#### Recovery Sessions

In a recovery session, a ‘virtual bird’ continues to recover until it has recovered all its caches. In reality, this is usually not the case; recovery percentages between 39% and 73% have been reported [8], [10], and another study mentions that ‘a few items were often cached and not recovered’ [12]. Furthermore, in some cases, the birds recovered relatively more of their ‘watched’ caches than their ‘in private’ ones [8], [10]. However, without a specific theory of scrub jay motivation – why they cache as much as they do, why they eat as much as they do – having the ‘virtual birds’ always recover everything seemed simplest.

Every time a ‘virtual bird’ must decide where to recover, it chooses the site with the highest ‘recovery attractiveness’ *R_k_*, as determined by Equation 5. Here, *F_k_* is a cache relocation effect, which helps the ‘virtual bird’ recover at sites where it has previously cached, while the inhibition of return *I_k_* and *noise* are the same as described previously, in Equations 2 and 3 respectively.
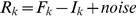
(5)The cache relocation effect *F_k_* associated with a site *k* depends on the existence of a cache chunk *o* referring to the same site. Then, the cache relocation effect associated with site *k* is equal to that chunk's current activation *A_o_* (Equation 1). Thus, the stronger a ‘virtual bird's’ memory of having cached somewhere, the more attractive it finds it to recover there.

If a ‘virtual bird’ recovers a worm, it needs to decide whether to re-cache it. To do so, it first calculates the *safety risk S_k_* associated with the site *k* where it just found its worm. This safety risk depends on its previous recovery experiences with site *k*'s tray, but only on those recovery experiences directed at its actual cache sites or their neighbors. This is in line with our previous work [21], where we show that scrub jays probably do not learn from *all* their recovery attempts, but only from those that are directed at sites where they have cached.

To calculate the safety risk *S_k_* associated with site *k*, a ‘virtual bird’ compares the ratio of ‘unsuccessful recovery attempts’ to ‘total recovery attempts’ within site *k*'s tray, according to Equation 6, where *A_u_* is the total activation of chunks encoding unsuccessful recovery attempts, and *A_s_* the total activation of chunks encoding successful recovery attempts. This ratio is then compared to the stress threshold *st*, which is a parameter that we tune (Table 1). If *S_k_* is greater than *st*, the ‘virtual bird’ re-caches the worm. Once a worm is re-cached in a recovery session, it is not recovered again. This assumption is made to keep the model as simple as possible, but it is also consistent with the empirical data. Unwatched, recovering scrub jays re-cache mainly in ‘out of tray’ locations, elsewhere in their home cages, and re-cache most worms only once [12].
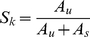
(6)


## Supporting Information

Figure S1 **Effect of different parameter values on the model's results.** Each panel is a summary of the model's results at different values for the noise parameter *n* and the decay parameter *d*, given a specific value for the stress threshold *st*. Grey circles mark parameter combinations that produced all five of the patterns listed in Table S2; white circles mark parameter combinations that produced four of the patterns or less.
(TIF)

Model Code S1 **Simulations implemented in a Java program,** ***CogCor*** **.**
(ZIP)

Table S1 **Range of parameter values evaluated.**
(DOCX)

Table S2 **Patterns present at different parameter combinations.**
(DOCX)

Text S1 **Robustness.**
(DOC)
