# Supplementary figures and images for: Corvid Re-Caching without ‘Theory of Mind’: A Model
Source: PLoS One. 2012 Mar 1;7(3):e32904. doi: 10.1371/journal.pone.0032904 (PMC3291480; doi:10.1371/journal.pone.0032904)

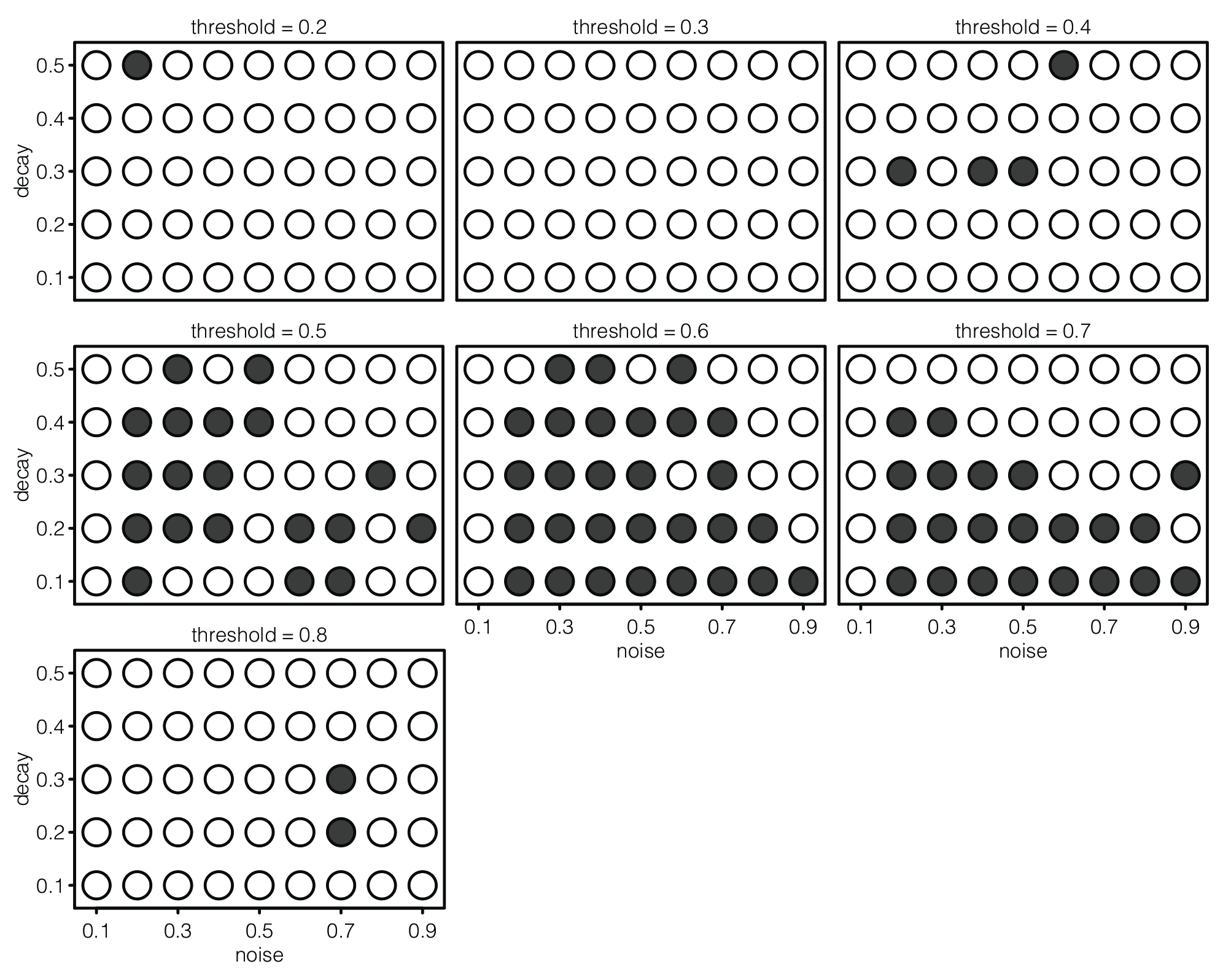

Supplement: Figure S1 — Effect of different parameter values on the model's results. Each panel is a summary of the model's results at different values for the noise parameter n and the decay parameter d, given a specific value for the stress threshold st. Grey circles mark parameter combinations that produced all five of the patterns listed in Table S2; white circles mark parameter combinations that produced four of the patterns or less. (TIF) [file pone.0032904.s001.tif]
