# Supplementary material for: Corvid Re-Caching without ‘Theory of Mind’: A Model
Source: PLoS One. 2012 Mar 1;7(3):e32904. doi: 10.1371/journal.pone.0032904 (PMC3291480; doi:10.1371/journal.pone.0032904)
Supplement: Table S1 — Range of parameter values evaluated. (DOCX) [file pone.0032904.s003.docx]

**Table S1. Range of parameter values evaluated.**

| **decay*, d*** | **noise*, n*** | **stress threshold*, st*** |
| --- | --- | --- |
| 0.1 – 0.5, by 0.1 | 0.1 – 0.9, by 0.1 | 0.2 – 0.8, by 0.1 |
