# Supplementary material for: Corvid Re-Caching without ‘Theory of Mind’: A Model
Source: PLoS One. 2012 Mar 1;7(3):e32904. doi: 10.1371/journal.pone.0032904 (PMC3291480; doi:10.1371/journal.pone.0032904)
Supplement: Table S2 — Patterns present at different parameter combinations. (DOCX) [file pone.0032904.s004.docx]

**Table S2. Patterns present at different parameter combinations.**

| **Experiment** | **Pattern** | **Percentage of Parameter Combinations with Pattern** |
| --- | --- | --- |
| 1: Watched  by a Conspecific | more re-caching in the ‘with onlooker’ condition^1^ | 89 |
| 1: Watched  by a Conspecific | more re-caching from the ‘with onlooker’ tray^1^ | 69 |
| 2: Onlooker Distance and Social Status | more re-caching with a dominant onlooker^2^ | 83 |
| 2: Onlooker Distance and Social Status | at least 20% more re-caching from the ‘near’ tray with a dominant onlooker^3^ | 54 |
| 2: Onlooker Distances and Social Status | at least 20% more re-caching from the ‘near’ tray with a subordinate onlooker^3^ | 61 |

^1^by Wilcoxon matched-pairs test; ^2^by Friedman’s analysis of variance; ^3^by direct numerical comparison
