# Supplementary material for: Corvid Re-Caching without ‘Theory of Mind’: A Model
Source: PLoS One. 2012 Mar 1;7(3):e32904. doi: 10.1371/journal.pone.0032904 (PMC3291480; doi:10.1371/journal.pone.0032904)
Supplement: Text S1 — Robustness. (DOC) [file pone.0032904.s005.doc]

**Supporting Information Text**

Corvid Re-Caching without ‘Theory of Mind’: A Model

**Authors and Affiliations**

Elske van der Vaart^1,2*^, Rineke Verbrugge^1^, and Charlotte K. Hemelrijk^2^

^1^Institute of Artificial Intelligence, ^2^Behavioural Ecology and Self-Organisation,

University of Groningen, Groningen, the Netherlands

*E-mail: e.e.van.der.vaart@rug.nl

**Robustness**

Our ‘virtual birds’ have three adjustable parameters for which we lack an empirical basis. These are decay *d*, which controls systematic loss of memory strength, noise *n*, which captures transient sources of error, and stress threshold *st*, which determines the proportion of recovery attempts that must be unsuccessful for the ‘virtual bird’ to start re-caching. All three of these parameters were tuned to produce a good fit of the empirical data. In order to systematically investigate the model’s sensitivity to their values, we ran a parameter search experiment. Within the range of values of Table S1, all possible combinations were tried. More extreme values of noise *n* or decay *d* were not considered, as they always resulted in recovery accuracy that was below chance level.

For each combination of parameter values, we studied 100 ‘virtual birds’ and noted whether they showed the same five patterns as the real birds (Table S2). It seems that our results are really sensitive only to the stress threshold (Figure S1). As long as that is kept between 0.5 and 0.7, our ‘virtual birds’ often behave similarly to the real birds [1,2]; otherwise, they do not. This makes sense: If the stress threshold drops too low, the ‘virtual birds’ re-cache virtually everything; if it is set too high, they hardly re-cache at all. In both cases, the model loses its sensitivity to differences between different trays, or to differences between different onlookers. Of course, if our account of scrub jay re-caching is correct, real birds must also experience some ‘psychological stress threshold’ at which they start to re-cache, so our model’s sensitivity to this parameter value seems realistic.

**References**

1. Emery NJ, Clayton NS (2001) Effects of experience and social context on prospective caching strategies by scrub jays. Nature 414: 443-446.

2. Dally JM, Emery NJ, Clayton NS (2006) Food-caching western scrub jays keep track of who was watching when. Science 312: 1662-1666.
